# Uncovering interactive effects of affective voice tone and personality diversity on dyadic creativity

**DOI:** 10.3389/fpsyg.2025.1668759

**Published:** 2025-11-24

**Authors:** Hiroyuki Sakai, Shigeo Yoshida, Tomosuke Maeda, Tomohiro Tanikawa

**Affiliations:** 1 Toyota Central R&D Labs., Inc., Nagakute, Aichi, Japan; 2 The University of Tokyo, Bunkyo, Tokyo, Japan

**Keywords:** creativity, emotion, personality, brainstorming, collaboration

## Abstract

Creativity is a key driver of innovation and social progress. Research on creativity has identified a variety of factors that affect creativity at both individual and group levels. However, the interactive effects of these factors in creativity have not been fully investigated. Thus, the present study aimed to explore the interactive effects of affective voice tone and personality traits on creativity in acquainted dyads. Pairs of participants took part in an experiment in which they cooperated on a verbal creativity task via a video conferencing system that modulated affective voice tone and completed personality questionnaires. The results demonstrate that affective voice tone modulation interacts with personality diversity to shape dyadic creativity. Specifically, while voice tone alone did not alter creative performance, it significantly modulated the positive effect of personality heterogeneity, suggesting that emotional vocal cues can constrain the benefits of interpersonal diversity during collaboration. This is the first empirical evidence for an interactive effect of affective voice tone and personality heterogeneity on dyadic creativity in close relationships. In addition, this study offers valuable insights into designing mechanisms and systems that enhance co-creation, not only in human teams but also in collaborations between humans and artificial intelligence agents.

## Introduction

Creativity is a key enabler of innovation and societal progress. Creativity is defined as the ability to create novel and useful ideas for problem solving (Runco and Jaeger, 2012). As well as being novel, creative ideas must also be useful in solving problems. Therefore, creativity is a multifaceted construct and encompasses divergent and convergent thinking processes, which work together to generate creative solutions (Guilford, 1950; Runco and Acar, 2012). Divergent thinking involves generating various original ideas, whereas convergent thinking refers to assessing and selecting ideas toward a single effective solution. Creative individuals approach problems from unique perspectives to devise creative solutions. By fostering creativity in various domains, such as science, technology and art, society can benefit from groundbreaking discoveries, revolutionary technologies, and cultural enrichment. Thus, understanding creativity has long remained a significant theme across various research fields, including psychology, biology, and sociology.

Over the past few years, team or group creativity has become increasingly important, as many creative outcomes in organizations emerge from collaboration rather than individual effort (Reiter-Palmon et al., 2012). Investigating group creativity is challenging because it involves a multitude of interacting variables. To comprehensively understand it, researchers must examine not only the creativity of the individuals comprising the group and the trait-level and state-level factors that influence it, but also clarify how their combinations and group communication processes interact to shape creative outcomes. The present study focused on disentangling part of these complex mechanisms that underlie group creativity.

Factors relating to creativity in intellectual activities involving multiple individuals have been researched. Brainstorming is a widely used technique for generating creative ideas through interpersonal communication. Sharing and exchanging ideas among members of a group is essential for successful brainstorming because exposure to ideas generated by others enhances creative performance at the group level (Dugosh et al., 2000; Fink et al., 2010, 2012; Paulus and Brown, 2007). Therefore, compared with more homogeneous groups, diverse groups are likely to show higher creative performance because group members can stimulate each other with a range of ideas from different perspectives. While numerous studies have examined the impact of group heterogeneity on creativity, their findings remain inconsistent. For example, some studies have shown positive effects of cultural diversity on group creativity (Li et al., 2017), but others have found negative effects (Gibson and Gibbs, 2006). According to a recent meta-analysis by Wang et al. (2019), deep-level diversity (e.g., knowledge, skill, ability) rather than surface-level diversity (e.g., age, sex, race/ethnicity) appears to play a beneficial role in group creativity (but see also Wallrich et al., 2024). Qi et al. (2022) further revealed that the creativity benefits of forging cognitively diverse work teams is mediated by team-level information elaboration. Group mood is considered as another important factor for group creativity. Group mood is defined as the aggregate of the moods of group members (George, 1990). It has been repeatedly demonstrated that positive group mood is positively associated with creative group performance (Huang and Huang, 2025; Huang et al., 2022; Kim and Shin, 2015; Shin, 2014; Shin et al., 2019). A different line of research has shown that positive group mood mitigates the negative effect of diversity in group member values (e.g., motivation, attitude) on creativity (Tang and Naumann, 2016). In contrast, findings on the effects of negative group mood are inconsistent. Although some studies have found a negative effect of negative group mood on group creativity (Huang et al., 2022; Shin, 2014; Tsai et al., 2012), others have shown its positive effect in some situations (Hoever et al., 2018).

Although the association between positive mood and creative performance has been repeatedly replicated, their causal relationship is less clear. Isen et al. (1987) conducted pioneering work on the causal role of stimulus-induced positive mood in creativity. They found that positive mood induced by comedy movies or small gifts facilitates creative ingenuity. This individual-level finding was replicated for group-level studies using recall of a recent past event of being in a good mood (Grawitch et al., 2003a,b). More recently, Sozo and Ogliari (2019) identified a set of stimuli that trigger positive mood, and demonstrated the facilitating effects of these stimuli on group creativity in an industrially practical task. Nonverbal affective cues can also be inducers of group mood (Vrugt and Vet, 2009). Using a videoconferencing system to modulate facial expressions in real time, Nakazato et al. (2014) showed that fake smiles improve the divergent aspect of dyadic creativity (i.e., the number of ideas generated). More recently, Nakada et al. (2025) demonstrated behavioral mimicry during avatar communication can have potential to enhance dyadic creativity. These lines of evidence suggest that affective interventions can augment creativity at both individual and group levels, although further research is needed to obtain a comprehensive view of this relationship. In particular, it is an intriguing question whether the induced positive mood itself or the exchange of accompanying affective cues is more crucial to group creativity. Indeed, Nakazato et al. (2014) found that while fake smiles enhanced dyadic creativity, they did not induce positive moods between the dyads, suggesting that exchanging positive affective cues rather than mood change itself is key. Likewise, in other intervention studies (Nakada et al., 2025; Sozo and Ogliari, 2019), it remains unclear whether positive mood changes actually occurred in the groups during creative tasks. Taken together, these findings raise an important question: to what extent can the exchange of affective cues explain variations in group creativity?

In the present study, we aimed to unravel the causal effect of affective tone of voice on dyadic creativity. This corresponded to the auditory version of the previous study (Nakazato et al., 2014) examining the effects of visual affective cue (i.e., smiles) on creativity. Voice is a powerful auditory cue that conveys affective information in communication (Scherer, 2003; Gobl and Chasaide, 2003). Affective prosody (variations in pitch, intensity, and timbre) serves as a primary channel for expressing affection and attitudes beyond verbal content, enabling listeners to infer speakers’ affective states (Cowen et al., 2019). Such paralinguistic signals dynamically shape interpersonal coordination during conversation (Ekstedt and Skantze, 2022). Affective tone rapidly biases social appraisals central to collaboration, including perceived trustworthiness (Belin et al., 2017; O’Connor and Barclay, 2017). These findings suggest that affective voice tone functions not merely as a vocal expression of affective states, but also as a direct modulator of interpersonal interaction. Therefore, our primary hypothesis was whether exchanging bottom-up affective information via voice tone alters performance in a cooperative creative task, and if so, whether it induced a positive mood. This point distinguished the present study from prior research that targeted the mood itself as the intervention (e.g., recall of good memories). In addition, compared to visual cues, auditory cues play a particularly central role in collaborative environments. In many remote or bandwidth-constrained communication settings, voice is the most stable and ubiquitous channel for conveying affective information. Thus, clarifying how voice tone influences creativity is not only relevant but also practically essential. This motivated our exploration of auditory affective cues, which are pervasive in remote collaboration settings.

Furthermore, we also examined the interaction between affective voice tone and individual differences in emotional contagion tendency. Previous research has shown that creative performance can be shaped by interactions between state-level and trait-level factors (Conner and Silvia, 2015), yet such effects have rarely been investigated in group creativity. In the current study, we focused on how the exchange of affective information through vocal tone may interact with the propensity to resonate with others’ emotional expressions. More specifically, we hypothesized that dyads with higher emotional contagion would be more responsive to affective vocal tone (Herrando and Constantinides, 2021), and consequently, its impact on creativity would be more pronounced. This hypothesis further extends our primary assumption regarding bottom-up affective modulation in collaborative creativity.

We also explored whether personality diversity boosts creativity and how such diversity effects might be influenced by voice tone modulation. In fact, there are a few studies showing the positive association between group-level creativity and personality diversity assessed based on the five-factor personality model (e.g., openness to experience: Schilpzand et al., 2011; extraversion: Barry and Stewart, 1997; Mohammed and Angell, 2004). Personality heterogeneity is generally expected to broaden cognitive search space, integrate multiple perspectives, and thereby enhance creativity (Wang et al., 2019). However, research on affective convergence suggests a potential counteracting force of such diversity benefits: when group members’ affective states become overly aligned, the productive tension arising from divergent viewpoints may diminish (van Kleef et al., 2017). In the context of the present study, such convergence could be reinforced by affective voice tone, potentially reducing the benefits of personality diversity. Therefore, we hypothesized that the positive relationship between personality diversity and collaborative creativity would be most evident under the neutral voice tone condition and would be attenuated under positive or negative tone conditions.

The present study therefore aimed primarily at examining the impact of affective voice tone on cooperative creative performance, while also allowing for exploratory examination of interaction of affective voice tone and personality heterogeneity. To this goal, we recruited acquainted pairs of participants and asked each dyad to cooperate on a verbal creativity task via a video conferencing system capable of modulating affective voice tone. While many experimental studies on interpersonal communication prefer unacquainted dyads to maximize control, we intentionally recruited participants who were already familiar with one another (friends, partners, or siblings). This decision was grounded in the ecological validity of the task. That is, real-world brainstorming and co-creation efforts often occur within pre-existing social relationships. The inclusion of acquainted dyads thus reflects a more naturalistic context for studying collaborative creativity. We also administered a questionnaire survey to assess the traits of each dyad. This is the first attempt to examine both the causal effect of affective voice tone and the effect of the interaction between affective voice tone and personality traits on dyadic creativity.

Taken together, the present study extends the literature on affective interventions in group creativity to the auditory modality by experimentally manipulating affective voice tone during real-time collaboration. In particular, if modulation of vocal tone does not bring about a noticeable change in group mood during collaboration, this would further support the notion that the exchange of bottom-up affective cues, rather than group mood itself, is sufficient to alter group creativity. Practically, understanding how voice tone shapes creative collaboration may inform the design of communication environments in organizations, such as remote teamwork, where the modulation of affective voice tone can be applied to foster collective creativity. This study therefore contributes to a more comprehensive model of how affective communication supports creativity at the interpersonal level.

## Materials and methods

### Participants

All participants were recruited in pairs of acquaintances. The inclusion criteria were (1) healthy adults in their 20 or 30 s, (2) ability to read, write, and communicate in Japanese, (3) not taking psychoactive drugs, and (4) not experiencing claustrophobia. All participants provided written informed consent. A total of 60 pairs (all Japanese) were enrolled in this study. There were 59 male participants (mean age = 26.1, standard deviation = 5.7 years) and 61 female participants (mean age = 27.5, standard deviation = 6.1 years). There were 18 male/male dyads, 19 female/female dyads, and 23 male/female dyads. Of the dyads, 36 were friends, seven siblings, and 17 partners (six married, 11 unmarried). The study protocol conformed to the Declaration of Helsinki and was approved by the institutional review boards of both University of Tokyo and Toyota Central R&D Laboratories, Inc.

### Apparatus

The experiment was carried out using a video conferencing system (Zoom; Zoom Video Communications, Inc., CA) on two laptop computers (MacBook Pro; Apple Inc., CA), and online whiteboard software (BuddyBoard; Brother Industries, Ltd., Nagoya, Japan) on two tablets (iPad; Apple Inc., CA). These devices were, respectively installed in two single-person soundproof booths. The video conferencing system transmitted the faces and voices of participants from one booth to the other. The voices were recorded using microphones, tone-modulated before transmission, and presented to participants via headphones. The whiteboard software enabled participants to share their answers with each other during the creative task.

The tone-modulation was performed using the audio processing software developed by Rachman et al. (2018). This software modulates voice tones in speech using real-time transformations of prosodic characteristics, such as pitch shifting, vibrato, inflection, and filtering. These effects are computationally efficient, ensuring less than 20 ms of latency. The software provides three affective voice tone modulation modes (i.e., happy, sad, afraid) through a combination of the real-time prosodic transformations. Validation studies confirmed that the modulated affective voice tone was recognizable and applicable across languages, including Japanese. In our experiment, we used the “happy” mode for positive tone modulation, the “sad” mode for negative tone modulation, and the “neutral” (no transformations) mode for a control condition.

### Procedure

After familiarizing themselves with the above-mentioned experimental setup, each dyad performed a cooperative version of the Divergent Association Task (DAT). In the original DAT (Olson et al., 2021), a participant is asked to name 10 single-word nouns that are as different from each other as possible within 4 min. The task performance (described below) has been demonstrated to be a reliable measure of divergent thinking ability, which is a key psychological component of creativity. In our task, the participant dyads were required to cooperatively perform the DAT for 15 min by communicating via the video conferencing system. This duration was determined in a small preliminary test to avoid excessive time pressure and to ensure sufficient opportunity for communication within dyads, which was in line with the study goal of observing collaborative interaction during idea generation. In the experiment, they were instructed to “brainstorm aloud” and reach consensus on exactly 10 words within the time limit. Conversation was free-form; no enforced turn-taking or speaking order was imposed. They were also asked to enter each agreed-upon noun on a shared online whiteboard and, if necessary, amend them as appropriate. During the 15-min task, either member could type candidate words, but the final list of 10 nouns was determined jointly through mutual verbal discussion, ensuring that both members consented to each selected word. The system did not automatically detect verbal agreement, and the experimenter did not intervene. Each dyad was blindly and randomly assigned to a positive (Happy), negative (Sad), or neutral (Neutral) tone-modulation condition. During the task, both participants’ voices were simultaneously and continuously modulated in real time, depending on the assigned condition. After the task, participants were asked to complete the NEO Five-Factor Inventory (Costa and McCrae, 1992), the Emotional Contagion Scale (ECS; Doherty, 1997), and a questionnaire assessing their overall impression of the experiment. The questionnaire consisted of four questions: (Q1: Comprehension) You understood the intention of the task correctly, (Q2: Enjoyment) You enjoyed the task, (Q3: Performance) You performed the task well, and (Q4: Uncomfortableness) You felt that there was something unusual about your partner’s voice. Each item was scored on a 4-point Likert scale (1 = strongly disagree, 2 = disagree, 3 = agree, 4 = strongly agree). These questionnaire responses were analyzed at the individual level rather than aggregated at the dyadic level.

### Creativity scoring

To measure creativity, responses on the DAT were assessed using an automated method. The creativity of responses in divergent thinking tasks is generally assessed manually by multiple trained raters (Amabile, 1983), so it is not always easy to obtain reliable scores (Silvia, 2008). In contrast, the DAT uses an automated scoring method based on a natural language processing technology (Olson et al., 2021). Specifically, the semantic distances (i.e., cosine distances) of all pairs of valid nouns are calculated using a machine learning language model and a creativity score is generated as the average of the semantic distances. In the present study, we first translated each noun into English and then calculated the average semantic distance using the GloVe model trained on the Common Crawl corpus (Pennington et al., 2014). In this regard, nouns that were not found in the GloVe vocabulary were treated as invalid and excluded from the calculation. Although semantic distance can alter depending on the language model used or culture and era in which the corpus was created, we adopted this scoring procedure for simplicity and ease of comparison, as recommended by the original study (Olson et al., 2021).

### Statistical analysis

We examined the effect of affective voice tone on creativity score using a between-subjects design. First, we used a one-way analysis of variance (ANOVA) to analyze DAT scores, with affective voice tone (Neutral, Happy, and Sad) as a between-subjects factor. Second, to examine the interactive effects of state and trait factors, we conducted an analysis of covariance (ANCOVA) with the pairwise mean of ECS scores as a covariate to incorporate the effect of the emotional contagion trait. We performed another ANCOVA to examine the interaction of affective voice tone (between-subjects factor) and dyadic personality heterogeneity (covariate). We used the pairwise cosine similarity of five-factor personality scores to quantify dyadic personality heterogeneity. Cosine similarity was selected because it reflects the relative configuration of trait profiles across all five dimensions, rather than focusing on absolute differences in trait levels. Compared with alternatives such as Euclidean distance or trait-wise variance, cosine similarity better captures whether two individuals exhibit similar or divergent overall personality structures, which is particularly relevant for examining how diversity in perspectives influences collaborative creativity. If the ANCOVA identified a significant interaction, we further performed a post hoc multiple comparison test to compare the regression slopes between the affective voice tone conditions. The significance threshold was set at *P* < 0.05.

In addition to the ANOVA and ANCOVA described above, we conducted a hierarchical multiple regression analysis to confirm the robustness of the observed effects and to address potential covariation among predictors. In this regression model, creativity scores were entered as the dependent variable, with condition (Neutral, Happy, Sad; dummy-coded with Neutral as the reference), dyadic personality heterogeneity, and ECS scores as main predictors. Four subjective task-impression ratings (Comprehension, Enjoyment, Performance, and Uncomfortable) were included as covariates of no interest. In the first step, all main effects were entered simultaneously. In the second step, two-way interactions between condition and trait factors were added to examine. Robust standard errors were used in all analyses. This regression analysis is reported in the Supplementary material.

## Results

**FIGURE 1:**
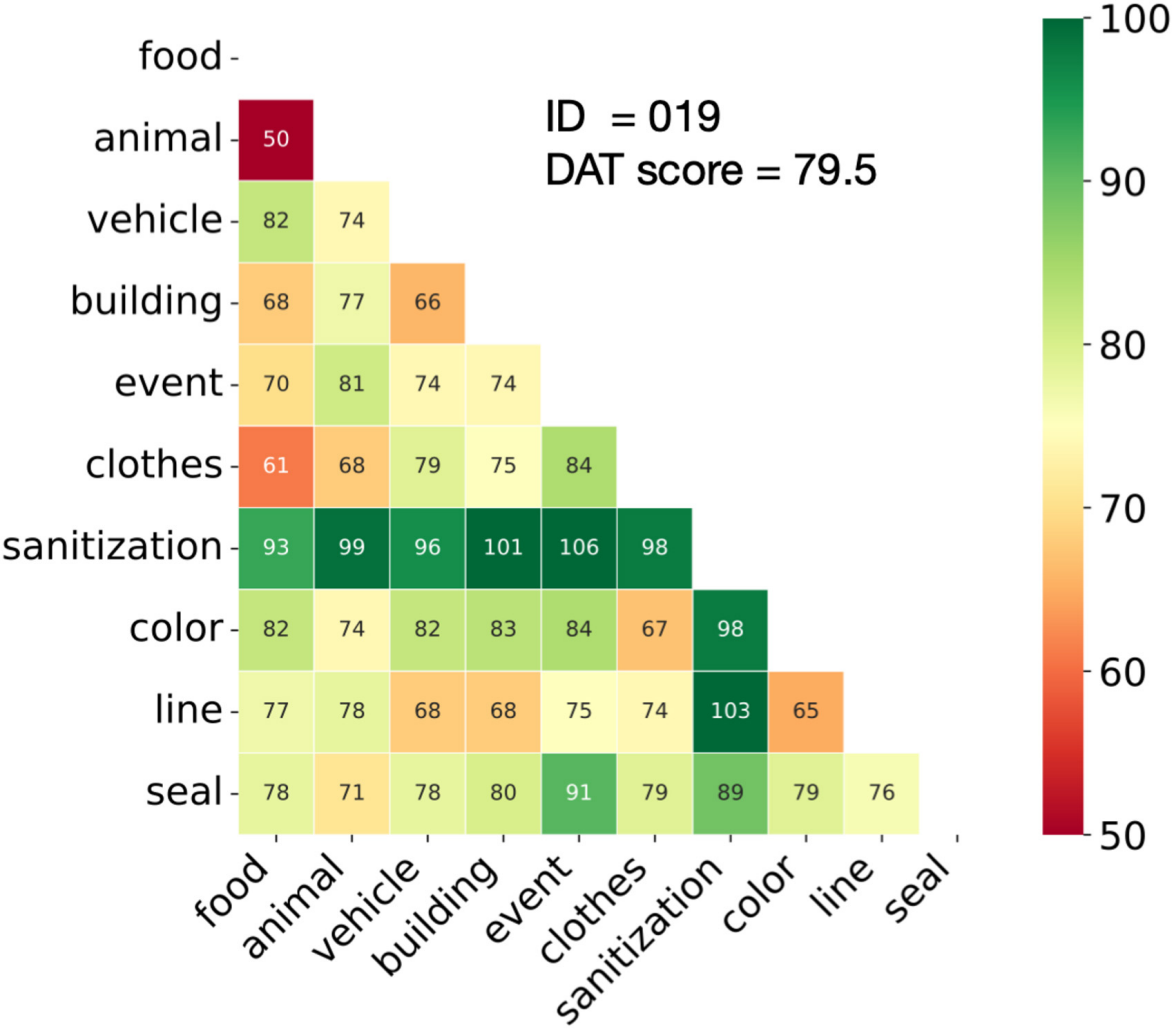
Association Task (DAT). For each participant dyad, the DAT score was computed as the average of the semantic distances of all pairs of responded nouns.

All the participant dyads completed the assigned tasks. The DAT scores ranged 70.4–91.1 with the mean score of 79.5 and the standard deviation of (SD) of 4.6, closely matching the distribution in the original study (Olson et al., 2021). Figure 1 shows example words and the corresponding DAT score from an average dyad.

**FIGURE 2:**
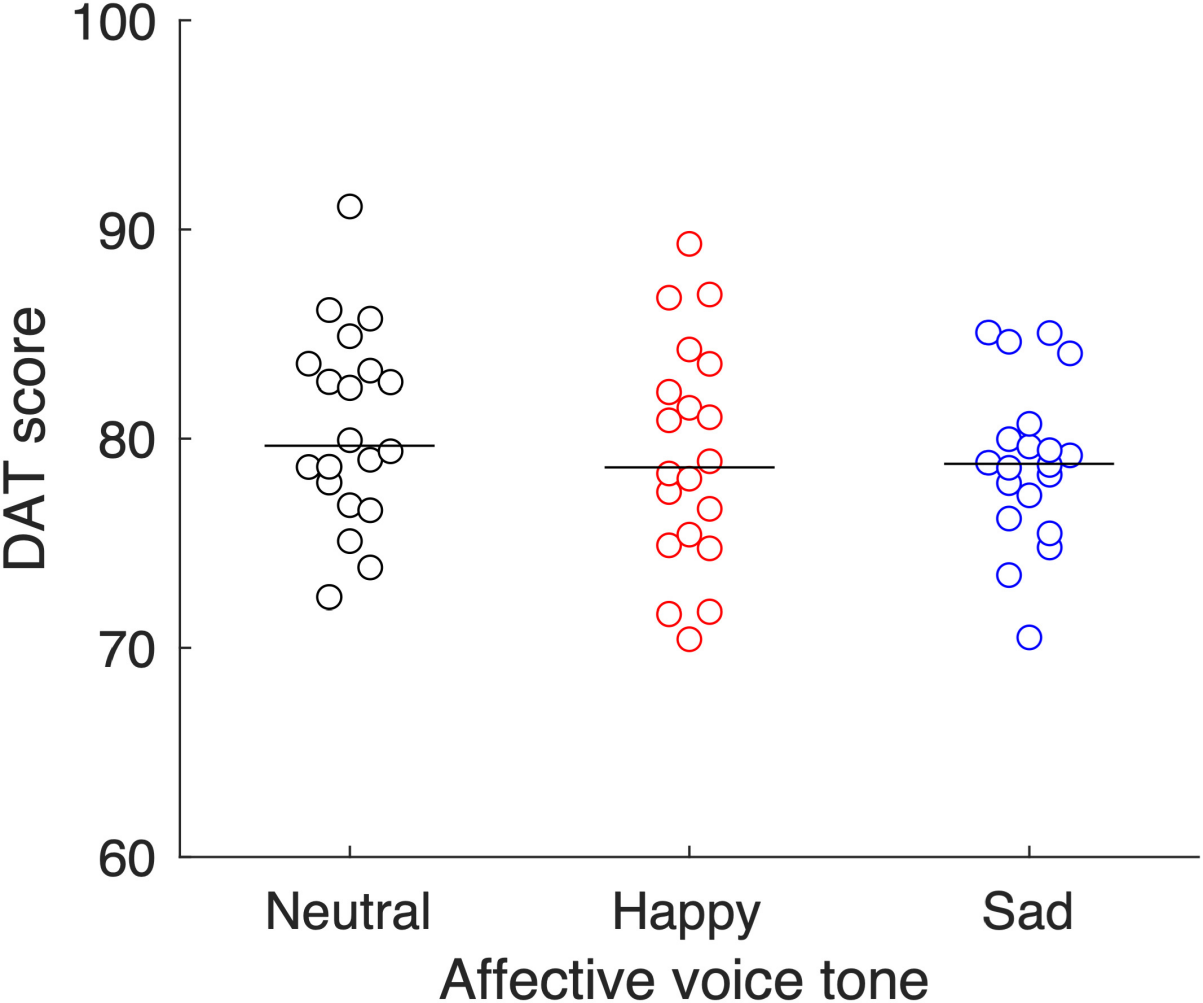
Effect of affective tone modulation on dyadic creative performance. Divergent Association Task (DAT) scores were compared among the three affective voice tone conditions. Each circle represents a dyad. The horizontal line indicates the mean value for each affective voice tone condition.

Figure 2 shows DAT scores as a function of the affective voice tone condition. The mean DAT scores were 80.5 (SD = 4.6) in the Neutral condition, 79.2 (SD = 5.3) in the Happy condition, and 78.8 (SD = 3.8) in the Sad condition, respectively. The one-way ANOVA of DAT scores showed no main effect for the affective voice tone condition (*F*_2, 57_ = 0.70, *P* = 0.50, η^2^ = 0.024).

**FIGURE 3:**
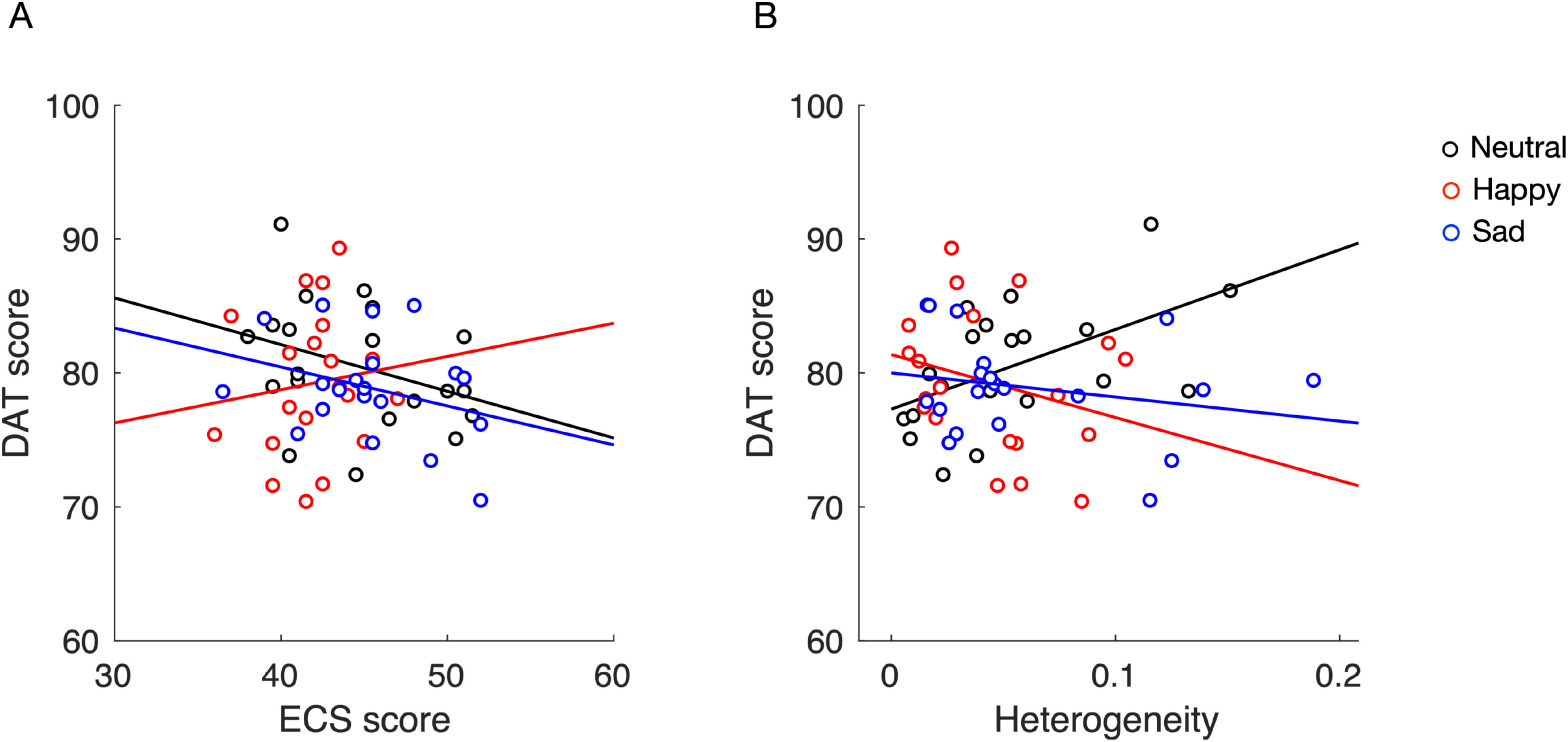
Interactive effects between affective tone modulation and personality traits on dyadic creative performance. Divergent Association Task (DAT) scores were examined using the Emotional Contagion Scale (ECS) score **(A)** or the personality heterogeneity **(B)** as a covariate. Each circle represents a dyad. The solid lines denote the affective voice tone-modulation condition. Different colors indicate different affective voice tone-modulation conditions.

When the ECS score was used as a covariate, the ANCOVA identified no significant interaction between affective voice tone and ECS score (*F*_2, 54_ = 0.89, *P* = 0.42, η_*p*_^2^ = 0.03). Nevertheless, visual inspection suggested a weak trend (Figure 3A), which may be worth exploring in future studies with larger samples. Specifically, there was a tiny positive correlation between DAT and ECS scores in the Happy condition (*r* = 0.12, 95% confidence interval (CI) [−0.34, 0.54], *P* = 0.60), but a weak negative correlation in the Neutral (*r* = −0.34, 95% CI [−0.68, 0.12], *P* = 0.14) and Sad (*r* = −0.32, 95% CI [−0.67, 0.15], *P* = 0.17) conditions. These results suggest that positive affective voice tone has a positive effect on dyadic verbal creativity, especially in dyads with higher emotional contagion. However, no definitive conclusions can be drawn from these results regarding the effect of affective voice tone on dyadic verbal creativity.

Interestingly, our analysis of dyadic personality heterogeneity identified a significant interaction with affective voice tone condition (*F*_2, 54_ = 4.42, *P* = 0.017, η_*p*_^2^ = 0.14; Figure 3B). The post hoc multiple comparisons indicated that the correlation between DAT score and dyadic personality heterogeneity in the Neutral condition was moderately positive (*r* = 0.54, 95% CI [0.12, 0.79], *P* = 0.015) and significantly greater than both those in the Happy (*r* = −0.28, 95% CI [−0.64, 0.19], *P* = 0.24) and Sad (*r* = −0.23, 95% CI [−0.61, 0.24], *P* = 0.33) conditions. These results suggest that affective voice tone modulation, whether positive or negative, suppresses the effect of personality heterogeneity on dyadic verbal creativity.

**FIGURE 4:**
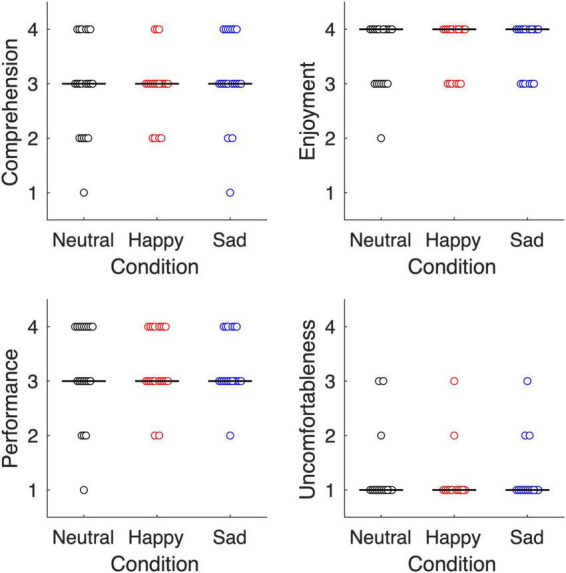
Participants’ overall impression of the experiment. Task comprehension, task enjoyment, task performance, and uncomfortableness when hearing partner’s voice were measured on a four-point Likert scale. Each circle represents a dyad. The horizontal line indicates the median value for each affective voice tone condition.

Figure 4 summarizes participants’ overall impression of the experiment. The Kruskal–Wallis test showed no significant differences in the metacognition of task comprehension (*χ*^2^ = 1.29, *P* = 0.53), task enjoyment (*χ*^2^ = 0.77, *P* = 0.68), and task performance (*χ*^2^ = 0.13, *P* = 0.94) among the affective voice tone conditions. More importantly, affective voice tone modulation did not have a significant effect on uncomfortableness with the voice (*χ*^2^ = 0.29, *P* = 0.87), suggesting that participants were unaware that their voices were modulated during the experiment.

To verify the robustness of these findings, we additionally performed a hierarchical multiple regression analysis including all main variables and covariates. The regression results reproduced the patterns observed in the ANOVA and ANCOVA (see Supplementary material for details).

## Discussion

In the current study, we investigated the interactive effects on affective voice tone and personality traits in dyadic creative performance of a collaborative creativity task. The results showed that affective voice tone modulation, regardless of its valence polarity, significantly suppressed the beneficial effects of personality heterogeneity on dyadic creativity. This is the first empirical evidence for an interactive effect of affective voice tone and personality heterogeneity on dyadic creativity in close relationships.

Contrary to our expectations, affective voice tone modulation per se did not have a strong effect on dyadic creativity. This result may suggest that inducing a positive group mood is indeed critical for enhancing group creativity performance (Huang et al., 2022; Kim and Shin, 2015; Shin, 2014; Shin et al., 2019) and that exchanges of bottom-up affective cues alone are insufficient. In fact, although a formal check of group mood was not conducted in this study, no significant differences in task enjoyment were observed among the affective voice tone conditions. This may indicate that the affective voice tone modulation employed in this study was insufficient to alter the group mood. However, as Nakazato et al. (2014) demonstrated using a visual affective cue, the induction of a positive group mood is not necessarily an essential factor in enhancing group creativity. Instead, there may be two alternative explanations for these unexpected results. First, the Neutral condition involved unmodulated voice transmission, which may have included naturally expressed affect. There is a possibility that (unmodulated) communication was conducted in a positive tone, particularly in acquainted dyads. In fact, the enjoyment ratings remained consistently high regardless of the modulation conditions. To rule out this possibility, it is necessary to use a modulation of the voice tone that is emotionally neutral. However, in co-creation tasks, which are often performed between acquaintances, it is ecologically valid for the baseline to be biased toward a somewhat positive mood. From this perspective, our results can be interpreted as indicating that voice tone modulation had no significant effect compared to natural communication between acquaintances. Second, continuous voice tone modulation may induce adaptation (Bestelmeyer et al., 2010; Nussbaum et al., 2022; Pinheiro et al., 2017). That is, when voice tone is always positive, it can quickly lose informational value as a social rewards, consequently weakening its impact on collaborative performance. Context-sensitive modulation of voice tone might therefore have a more potent effect on collaborative creativity.

Moreover, the modulation effect due to emotional contagion trait also failed to meet our expectation. Several factors may account for this null finding. First, statistical power was limited in the present study. In fact, a weak but positive correlation was observed between emotional contagion trait and dyadic creative performance only in the positive modulation condition (Happy); this was in contrast to the negative correlations found in the other two conditions (Neutral and Sad). These tendencies appear consistent with the view that positive affective voice tone tends to enhance dyadic creativity in more emotionally contagious dyads. Second, variance in ECS may have been restricted in our sample of acquainted pairs, reducing the sensitivity to detect moderation. Third, the impact of personality heterogeneity, which produced a robust interaction with tone, may have overshadowed subtler ECS-related effects. Taken together, these considerations suggest that while emotional contagion trait may remain theoretically relevant, its influence may be more detectable in larger or more heterogeneous samples.

Affective voice tone modulation influenced dyadic creativity in a more complex way. Only in the Neutral condition, dyadic creativity was positively associated with personality heterogeneity, indicating that in the absence of affective voice tone modulation, more diverse dyads tended to show higher creative performance. This is consistent with previous studies on the effects of personality on group creativity. For example, Schilpzand et al. (2011) demonstrated that the standard deviation, but not the mean, of the openness to experience trait in group members was positively associated with group creativity. Findings from other studies indicate that greater variance in the extraversion trait favors group creativity (Barry and Stewart, 1997; Mohammed and Angell, 2004). These findings as well as our own suggest that individuals with more diverse personality can provide more creative ideas from different perspectives when interacting in brainstorming situations. While the present study quantified personality diversity using cosine similarity as an overall multivariate index based on the five-factor model, future research could examine whether diversity in specific traits or alternative indices of trait diversity yield similar patterns. In particular, the relationship between the diversity of meta-traits derived from the Big Two model (Feist, 2019) and group creativity remains an intriguing question that has yet to be investigated. Such analyses would help identify which aspects of personality composition most strongly contribute to collaborative creativity.

The present study is distinct in demonstrating that affective voice tone modulation may attenuate the association between personality heterogeneity and dyadic creative performance. This suggests a potential masking effect of affective expression through voice tone on cooperative creative performance, particularly in individuals with diverse personality. More generally, affective feedback during cooperative creation may inhibit the generation of diverse ideas from the different perspectives that reflect personality diversity. Previous research has shown that cognitive diversity can be beneficial for creativity by encouraging cognitive conflicts during the integration of divergent viewpoints, such as idea stimulation, perspective exchange, and constructive disagreement (Li et al., 2017; Wang et al., 2019). When affective convergence reduces such conflicts, it may inadvertently suppress the very mechanisms that make diversity valuable in collaborative creativity. Another possible interpretation is that the constant affective tone, whether positive or negative, might have obscured spontaneous vocal reactions to each other’s suggestions, such as emphasis, hesitation, or excitement. Such reactions can signal evaluation and foster interactive debate. In contrast, the unaltered voice in the Neutral condition allowed context-dependent variations in prosody, potentially preserving the adaptive tension that stimulates creativity. This unexpected interaction between affective voice tone modulation and personality diversity raises an intriguing question. One potential explanation is that affective voice tone may excessively promote affective convergence and consequently reduce social friction. While reducing social friction may increase interpersonal comfort during communication, it could also constrain the divergent/conflicting perspectives typically afforded by personality heterogeneity. However, this mechanism was not directly measured in the present study and remains speculative. Future research should examine whether affective convergence indeed mediates the observed interaction.

The present findings have implications not only for the general understanding of dyadic creative performance, but also for the development of artificial intelligence (AI) to promote human creation. With the rapid development of large language models, co-creation between humans and AI using dialogue systems is becoming a reality (Chiou et al., 2023; Filippi, 2023). Currently, humans rely solely on textual information to communicate with AI systems. However, the identification of nonverbal communication factors involved in human collaborative creativity and the incorporation of such factors into AI avatars could further promote co-creation (Nakada et al., 2025). In particular, our findings suggest that continuous, non-contextual affective expression through voice tone increases the user’s sense that the system is a social actor, which can shift norms toward harmony and reduce disagreement, thereby dampening diversity benefits in cooperative creation. Therefore, the present findings motivate that AI avatars may need to avoid sustained positive socio-emotional cues in brainstorming settings. In addition, it may also be essential to maximize the diversity benefits by establishing prompts to generate AI personalities that deviate from the user’s personality. In fact, Serapio-García et al. (2023) proposed a simple method for shaping the personality of widely-used large language models, without any additional learning. These are fascinating directions for future research in the era of AI, because AI-human cooperation has great potential as a new intelligent entity (Sakai et al., 2024).

The present study had several limitations. First, the sample size per condition (20 dyads) was relatively small, which may have limited statistical power and reduced the ability to detect subtle effects or interactions. Future studies with larger and more diverse samples will be important to validate and extend the current findings. Second, only one verbal creative task (i.e., the DAT) was used to measure dyadic creativity. The DAT captures semantic dispersion within the responded words and thus indexes a specific facet of divergent thinking. It does not assess creativity ratings by human judges or problem-solving under constraints. We therefore explicitly acknowledge the limited scope of the DAT and the need for a broader assessment battery for future work. We also believe it is important to replicate the current findings in more practical creativity settings. Third, we did not include a formal manipulation check to confirm whether affective voice modulation induced the intended group mood. This limitation is particularly important because it leaves open the question of whether the current findings necessitate participants’ conscious awareness of group mood. Fourth, each dyad was exposed to the same affective voice tone modulation. According to a recent study by Kung and Chao (2018), interpersonal expression of mixed emotions plays an facilitative role in identifying creative solutions in a negotiation task. Mixed affective voice tone modulation (e.g., Happy for one member and Sad for another) may have a greater effect on dyadic creativity (To et al., 2021). Fifth, in the present study, demographic and relational variables (e.g., closeness, communication patterns) across dyads were not systematically balanced across voice tone conditions. These factors may have introduced uncontrolled variability in both interpersonal dynamics and creative behavior. Although participants were randomly assigned to conditions, future research with stratified or fully counterbalanced designs will be necessary to isolate the effects of these variables more precisely.

## Conclusion

This study provides the first empirical evidence that affective voice tone interacts with personality diversity to shape dyadic creativity in acquainted pairs. While affective voice modulation alone did not significantly enhance creative performance, it diminished the creativity benefits typically afforded by personality heterogeneity. This unexpected interaction suggests that affective cues may foster implicit affective convergence, which can hinder diverse perspectives essential for collaborative ideation. By focusing on acquainted dyads, this research offers ecologically valid insights into how affective communication dynamics operate in creative collaboration. These findings not only contribute to our understanding of human co-creativity but also offer design implications for emotionally expressive systems, including AI avatars in collaborative environments. Future research should investigate broader demographic configurations and alternative affective strategies to refine mechanisms that enhance, rather than constrain, creativity in diverse dyads, as well as humans and AI agents.

## Data Availability

The raw data supporting the conclusions of this article will be made available by the authors, without undue reservation.
