# Supplementary material for: Uncovering interactive effects of affective voice tone and personality diversity on dyadic creativity
Source: Front Psychol. 2025 Nov 24;16:1668759. doi: 10.3389/fpsyg.2025.1668759 (PMC12684382; doi:10.3389/fpsyg.2025.1668759)
Supplement: Supplementary file 1 [file Data_Sheet_1.pdf]

Supplementary Material

S1. Hierarchical Regression Analysis

Method

To verify the robustness of the effects observed in the main analyses (ANOVA/ANCOVA), a hierarchical multiple regression analysis was conducted with creativity scores as the dependent variable. All predictors were mean-centered, and robust standard errors were used. In Step 1, main effects of the experimental condition (dummy-coded with Neutral as the reference level), dyadic personality heterogeneity, emotional contagion trait (i.e, ECS score), and four control variables assessing participants' subjective task impressions (Comprehension, Enjoyment, Performance, Uncomfortable) were entered simultaneously. In Step 2, two-way interactions between condition and each of the two trait factors (personality heterogeneity, emotional contagion) were added to examine whether the state manipulation (affective voice tone) moderated the effects of the trait factors.

Results

Step 1: The model was not significant overall, $F(8, 51) = 1.02$, $p = .431$, with an R^2 of .14. All variance inflation factors (VIFs) were below 2.5, indicating no problematic multicollinearity. No main effect of condition was observed (both Happy and Sad vs. Neutral, *ps* > .27). Neither personality heterogeneity nor emotional contagion tendency significantly predicted creativity in this model (*ps* > .08).

Step 2: When interaction terms were added, the model became significant overall, $F(12, 47) = 2.33$, $p = .020$, with $R^2 = .32$. All VIFs were below 3.5, indicating no problematic multicollinearity. For emotional contagion tendency, none of the simple slopes reached significance (*ps* > .08), although there was a slight negative trend in the Neutral condition ($\beta = –0.36$, 95% CI [–0.76, 0.04], $p = .086$). Meanwhile, for personality heterogeneity, a clear moderation effect of condition emerged. In the Neutral condition, personality heterogeneity positively predicted creativity ($\beta = 64.4$, 95% CI [8.2, 120.6], $p = .029$). In the Happy condition, this slope became negative but nonsignificant ($\beta = –43.1$, 95% CI [–99.4 13.2], $p = .14$). In the Sad condition, the slope was also negative and nonsignificant ($\beta = –14.0$, 95% CI [–67.0 38.9], $p = .61$). Thus, the beneficial effect of personality diversity on dyadic creativity was observed only in the Neutral condition, and it diminished under both affective voice tone manipulations. These results were consistent with the ANOVA/ANCOVA results.
